# Emerging Trends in Toxoplasmosis Seroepidemiology in Childbearing-Aged Women in Croatia, 2015–2024

**DOI:** 10.3390/pathogens14080796

**Published:** 2025-08-08

**Authors:** Mario Sviben, Klara Barbić, Maja Bogdanić, Ema Reicher, Sara Glavaš, Dan Navolan, Ana Sanković, Tomislav Meštrović, Ivan Mlinarić, Simona Vlădăreanu, Radu Vlădăreanu, Tatjana Vilibić-Čavlek

**Affiliations:** 1 Department of Parasitology, Croatian Institute of Public Health, 10000 Zagreb, Croatia; mario.sviben@hzjz.hr; 2 School of Medicine, University of Zagreb, 10000 Zagreb, Croatia; 3 Statistics Concentrator, Harvard University, Cambridge, MA 02139, USA; klarabarbic@college.harvard.edu; 4 Department of Virology, Croatian Institute of Public Health, 10000 Zagreb, Croatia; maja.bogdanic@hzjz.hr (M.B.); sara.glavas@hzjz.hr (S.G.); 5 Department of Clinical Microbiology, University Hospital for Infectious Diseases “Dr. Fran Mihaljević”, 10000 Zagreb, Croatia; ema.reicher@gmail.com; 6 Department of Obstetrics and Gynecology, “Victor Babes” University of Medicine and Pharmacy, 300041 Timisoara, Romania; navolan@umft.ro; 7 Department of Microbiology, University of Applied Health Sciences, 10000 Zagreb, Croatia; asankovic@outlook.com; 8 University Centre Varaždin, University North, 42000 Varaždin, Croatia; tmestrovic@unin.hr; 9 Institute for Health Metrics and Evaluation and the Department of Health Metrics Sciences, University of Washington, Seattle, WA 98195, USA; 10 Department of Epidemiology, Croatian Institute of Public Health, 10000 Zagreb, Croatia; ivan.mlinaric@hzjz.hr; 11 Neonatology Clinic Elias, Department of Obstetrics-Gynecology and Neonatology, “Carol Davila” University of Medicine and Pharmacy, 050474 Bucharest, Romania; simconst69@gmail.com; 12 Obstetrics and Gynecology Clinic Elias, Department of Obstetrics and Gynecology, “Carol Davila” University of Medicine and Pharmacy, 050474 Bucharest, Romania; vladareanu@gmail.com

**Keywords:** *Toxoplasma gondii*, epidemiology, childbearing-aged women, Croatia

## Abstract

Childbearing-aged and pregnant women represent a risk group for *Toxoplasma gondii* infection due to possible transplacental transmission resulting in congenital toxoplasmosis. We analyzed the seroepidemiological trends of toxoplasmosis in Croatia over ten years (2015–2024). A total of 2791 childbearing-aged and pregnant women were included. *Toxoplasma gondii*-specific IgM/IgG antibodies were detected using an enzyme-linked fluorescence assay. Samples with positive IgM and IgG antibodies were tested for IgG avidity. IgG antibodies were detected in 695 (24.9%) participants, while acute toxoplasmosis (IgM antibodies and low avidity IgG antibodies) was confirmed in 32 (1.2%) of participants. The IgG seroprevalence showed a declining trend over the years. Residents of suburban/rural areas were more often seropositive than those in urban areas (31.4 vs. 22.3%). Logistic regression analysis revealed that year of testing, age, and settlement were associated with the risk of seropositivity. For each later calendar year, the log odds of being IgG-positive decreased, while for each additional year of age, the log odds increased. Residence in an urban area was associated with lower log odds. The region was not a significant predictor in the logistic regression. The differences in seropositivity observed across regions can be mainly attributed to Pannonian Croatia, which showed significantly higher odds of IgG seropositivity. Data about the toxoplasma serological status is useful for planning prevention campaigns.

## 1. Introduction

*Toxoplasma gondii* is an obligate intracellular protozoan parasite of medical and veterinary importance. Its life cycle includes a sexual stage that occurs in the intestinal epithelium of cats and an asexual phase that involves different warm-blooded animals. *Toxoplasma gondii* exists in two morphologic forms: tachyzoites, rapidly dividing cells, and bradyzoites, slow-replicating, latent form of the parasite found within tissue cysts. Human infections occur through the ingestion of food or water contaminated with oocysts containing sporozoits from cat feces, or by consuming raw or undercooked meat containing tissue cysts [1]. Vertical transmission may occur after the transplacental spread of tachyzoites in pregnant women with a primary infection during pregnancy, leading to congenital toxoplasmosis. Reactivations of latent toxoplasmosis and reinfections by another strain may also occur [2].

Toxoplasmosis is considered to be the most prevalent human parasitic infection. Globally, over 60% of some populations are seropositive to *T. gondii*. The seroprevalence rates vary between regions. Infection rates tend to be higher in hot and humid tropical areas, as oocysts develop more rapidly and survive better in these conditions [3].

In more than 80% of immunocompetent individuals, toxoplasmosis is asymptomatic. In symptomatic cases, it is a mononucleosis-like disease characterized by fever, headache, malaise, and tender lymphadenopathy [4]. Severe disease, including disseminated infections with fatal outcome, has been reported in immunocompetent individuals with acute toxoplasma infections in certain tropical regions, caused by atypical, more virulent *T. gondii* strains associated with a high parasite load [5,6]. In immunocompromised patients, reactivation of latent toxoplasmosis may cause encephalitis, chorioretinitis, pulmonary, or disseminated toxoplasmosis [7,8,9].

Congenital toxoplasmosis results from the *T. gondii* transplacental transmission following maternal infection acquired during pregnancy [10]. The incidence of congenital toxoplasmosis depends on the trimester in which the maternal infection was acquired. The likelihood of the maternal-fetal transmission rate increases with gestational age at the time of maternal seroconversion, from approximately 25% in the first trimester to 65% in the third trimester. Conversely, if the maternal infection occurs later in pregnancy, the likelihood of symptomatic congenital infection decreases (infections contracted during the third trimester are typically asymptomatic at the time of birth) [11]. Congenital toxoplasmosis can present with a wide range of clinical symptoms, from asymptomatic infection to severe neurological and ocular disease. About 75% of infants with congenital toxoplasmosis show no obvious clinical manifestations at birth [3]. Symptoms of congenital toxoplasmosis include intracerebral calcifications, chorioretinitis, hepatosplenomegaly, jaundice, and maculopapular rash [12]. Infants with subclinical or mild infection are at risk of developing late sequelae, including motor delays, learning disorders, and hearing loss [3].

The epidemiology of toxoplasmosis differs regionally. A recent review analyzing toxoplasma seroprevalence across 30 European countries between 2000 and 2020 reported an overall anti-*T. gondii* IgG antibody prevalence of 32.1%, with significant variations observed between countries. Northern Europe had the lowest prevalence at 20.1%, while Western, Eastern, and Southern Europe showed higher rates of 38.5%, 39.7%, and 27.5%, respectively [13]. In the subgroup of childbearing-aged and pregnant women, the seropositivity varied from 9.31% to 24.64% (northern regions) [14,15]; 31.70% to 48.66% [16,17] (western regions); 15.97% to 52.09% [18,19] (eastern regions) and 17.89% to 48.58% (southern regions) [20,21].

The diagnosis of toxoplasmosis in humans is mainly based on the detection of specific IgM and IgG antibodies [22]. Positive toxoplasma IgM antibodies are commonly considered as an indicator of acute or recent infection, while IgG antibodies indicate previous infection. However, since IgM antibodies can remain detectable for months or even years after the primary infection, distinguishing between an acute and a past infection can be challenging [23]. Determination of IgG avidity in these cases can differentiate recent (low avidity antibodies) from previous infection (high avidity antibodies) [24].

Seroprevalence studies conducted among childbearing-aged and pregnant women in Croatia have shown an overall toxoplasma seropositivity rate of 29.1% (2005–2009) [25] and 20.1% from 2014 to 2023 [26]. A regional study conducted in 1994–1995 found a seropositivity rate of 38.1% among the female population in Split-Dalmatia County [27]. However, no studies analyzed the seroprevalence trends in this population group.

Given the public health significance of toxoplasmosis as a TORCH infection and possible severe outcomes in infected children, this large-scale seroprevalence study aimed to analyze the seroepidemiology trends of toxoplasmosis among Croatian women of childbearing age over ten years (2015–2024).

## 2. Materials and Methods

### 2.1. Characteristics of Study Participants

The study included 2791 childbearing-aged and pregnant women of Croatian nationality aged 16–45 years, who were consecutively tested as a part of routine TORCH profile between January 2015 and December 2024 at the Croatian Institute of Public Health, the largest public health institution in the country.

For this study, participants were classified based on age (five-year age groups), settlement type, and geographic region. *Toxoplasma gondii* IgM and IgG prevalence rates were analyzed in two time periods (2015–2019 and 2020–2024) and yearly. No significant difference in age was observed between years (*p* = 0.061), with a median age ranging from 31 (interquartile range; IQR = 26–36) to 33 (IQR = 28–38) years.

**Figure 1:**
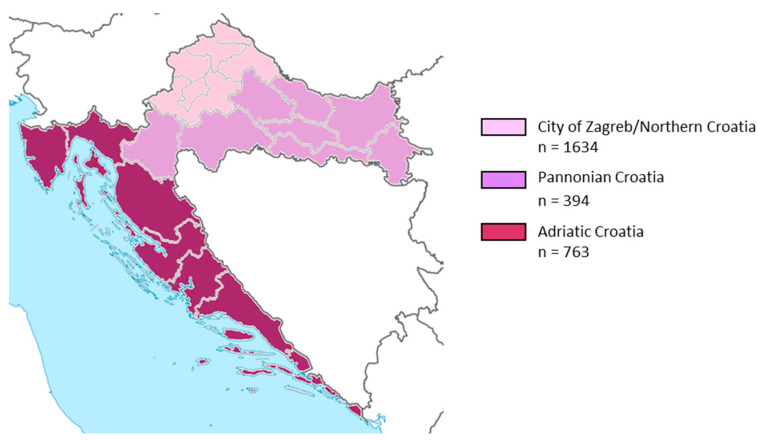
Distribution of study participants by geographic region.

To analyze the regional seroprevalence, according to the Nomenclature of Territorial Units for Statistics (NUTS) of the European Union, three geographic regions were defined: Pannonian Croatia, Adriatic Croatia, and the City of Zagreb/Northern Croatia [28]. The majority of participants were residents of continental areas: City of Zagreb/Northern Croatia (*n* = 1634; 58.6%) and Pannonian Croatia (*n* = 394; 14.1%), while 763 (27.3%) of participants were from Adriatic Croatia (Figure 1).

In all tested years, the majority of participants were from urban areas (overall 1994; 71.4%, range 55.4–83.7%).

### 2.2. Serological Testing

Serum samples were collected and tested for IgM and IgG antibodies to *T. gondii*. All IgM/IgG-positive samples were further tested for IgG avidity to confirm/rule out recent infection. Serological tests were performed using automated enzyme-linked fluorescence assays (ELFA; Vidas TOXO IgM/IgG/IgG avidity, Biomerieux, Marcy-l’Étoile, France) and interpreted as follows: IgM index < 0.55 negative, 0.55–0.65 borderline, >0.65 positive; IgG IU/mL < 4 negative, 4–8 borderline, >8 positive; IgG avidity index < 0.3 low (acute/recent infection), 0.3–0.5 borderline, >0.5 high (past infection).

### 2.3. Statistical Analysis

Descriptive statistics were used to summarize the distribution of participants’ age, geographic region, and settlement type across the study period.

Prevalence estimates were reported as percentages with corresponding 95% confidence intervals (CI), computed using binomial approximation methods.

To analyze seroprevalence trends by age group, participants were categorized into six age intervals, and IgG prevalence with exact binomial 95% CI was calculated for each year. Differences in prevalence between age groups were assessed using Pearson’s chi-square test for independence, with *p*-values reported for each annual comparison.

A multivariable logistic regression model was used to identify independent predictors of IgG seropositivity, including age (continuous), year, geographic region, and settlement type. Interaction was tested between age and each of the other variables to assess effect modification. Regression results were presented as log odds estimates with 95% CI, and model outputs were visualized using coefficient plots. Predicted probabilities of IgG seropositivity were derived from the final model and stratified by settlement type and region using marginal effects plots generated with the ggeffects package. All figures were produced using the ggplot2 package in R.

All analyses were two-sided, and a *p*-value < 0.05 was considered statistically significant. Statistical analysis was performed using R software (version 4.4.2, R Foundation for Statistical Computing, Vienna, Austria).

## 3. Results

### 3.1. Toxoplasma gondii Seroprevalence Trends from 2015 to 2024

**Figure 2:**
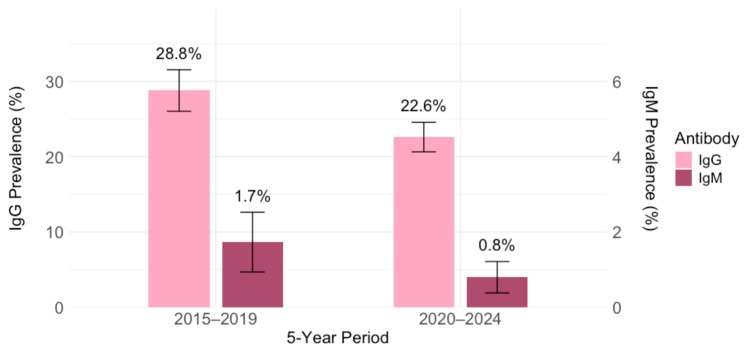
Prevalence of *Toxoplasma gondii* IgM and IgG antibodies in five-year intervals: 2015–2019 and 2020–2024 (% positive with 95% confidence intervals).

*Toxoplasma gondii* IgG antibodies were detected in 695/2791 (24.9%; 95% CI = 23.3–26.9) participants. Comparing the IgG seropositivity rates in 2015–2019 with those in 2020–2024, a significant decline in seroprevalence was observed (*p* < 0.001) (Figure 2). In the first five-year period, the seroprevalence rate was 28.8% (299/1039; 95% CI = 26.0–31.6), ranging from 21.4% (95% CI = 15.6–28.1; 2017) to 34.8% (95% CI = 28.3–41.8; 2018), and in the second five-year period 22.6% (396/1752; 95% CI = 20.7–24.6), ranging from 20.3% (95% CI = 15.2–26.2; 2021) to 25.6% (95% CI = 19.2–32.9; 2020).

*Toxoplasma gondii* IgM antibodies were detected in 53 (1.9%) participants on initial screening (ELFA). Acute/recent toxoplasmosis was confirmed by low or borderline avidity in 32 (1.2%; 95% CI = 0.8–1.6) participants. The overall IgM prevalence was higher in the 2015–2019 period than in 2020–2024 (18/1039; 1.7%; 95% CI = 1.0–2.7 vs. 14/1752; 0.8%; 95% CI = 0.4–1.3). Prevalence of acute toxoplasmosis ranged from 1.0% (95% CI = 0.1–3.4; 2019) to 2.8% (95% CI = 1.0–6.0; 2016) in the first time-period and from 0.2% (95% CI ≤ 0.1–1.2; 2023) to 2.4% (95% CI = 0.6–6.0; 2020) in the second time-period (Figure 2). These temporal differences were statistically significant (*p* = 0.025).

**Figure 3:**
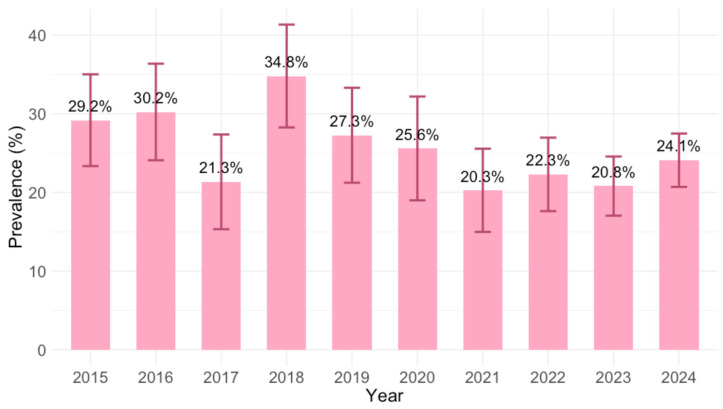
Prevalence of *Toxoplasma gondii* IgG antibodies by year (% positive with 95% confidence intervals).

The IgG seroprevalence varied significantly between years (*p* = 0.002), ranging from 20.3% (95% CI = 15.2–26.2; 2021) to 34.8% (95% CI = 28.3–41.8; 2018). Analyzing the yearly IgG prevalence, except 2017 (21.3%, 95% CI = 15.6–28.1), a declining trend was observed from 2018 (34.8%; 95% CI = 28.3–41.8) to 2021 (20.3%; 95% CI = 15.2–26.2), with a stable trend afterward (Figure 3).

**Figure 4:**
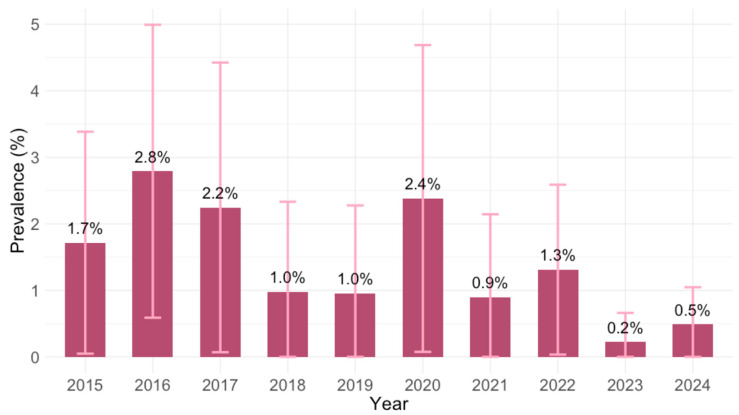
Prevalence of *Toxoplasma gondii* IgM antibodies by year (% positive with 95% confidence intervals).

Analyzing the yearly IgM prevalence, seropositivity ranged from 0.2% (95% CI ≤ 0.1–1.2; 2023) to 2.8% (95% CI = 1.0–6.0; 2016) (Figure 4). These differences were of borderline significance (*p* = 0.068).

### 3.2. Toxoplasma gondii IgG Seroprevalence by Age

**Figure 5:**
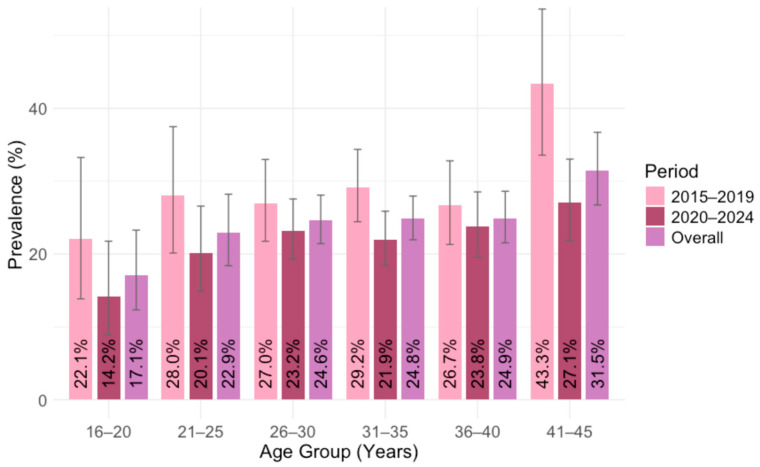
*Toxoplasma gondii* IgG seroprevalence by age in two time periods: 2015–2019 and 2020–2024 (% positive with 95% confidence intervals).

Analyzing the overall IgG seroprevalence by age, significant differences were found between age groups (*p* = 0.014). The lowest seropositivity was observed in the 16–25–year group (17.1%; 95% CI = 12.3–23.3). Thereafter, seroprevalence was stable, ranging from 22.9% (95% CI = 18.4–28.2) in the 21–25-year group to 24.9% (95% CI = 21.5–28.6%) in the 36–40-year group. The highest seropositivity was in the 41–45-year group (31.5%; 95% CI = 26.7–36.7) (Figure 5).

Comparing the IgG seroprevalence in two time periods, significant differences in the seropositivity between age groups were observed in the first period. In 2015–2019, seroprevalence rates ranged from 22.1% (95% CI = 13.8–33.3) to 43.3% (95% CI = 33.6–53.6; *p* = 0.039). However, no significant differences were detected in 2020–2024, ranging from 14.2% (95% CI = 8.9–21.8) to 27.1% (95% CI = 21.6–33.2; *p* = 0.133) (Figure 5).

The yearly IgG prevalence by age groups is presented in Table 1. Analyzing the seroprevalence by year, a significant difference was observed only in 2018, ranging from 15.4% to 50.0% (*p* = 0.026), while in 2016 it was of borderline significance (*p* = 0.098).

### 3.3. Toxoplasma gondii IgG Seroprevalence According to Geographic Region and Settlement

**Figure 6:**
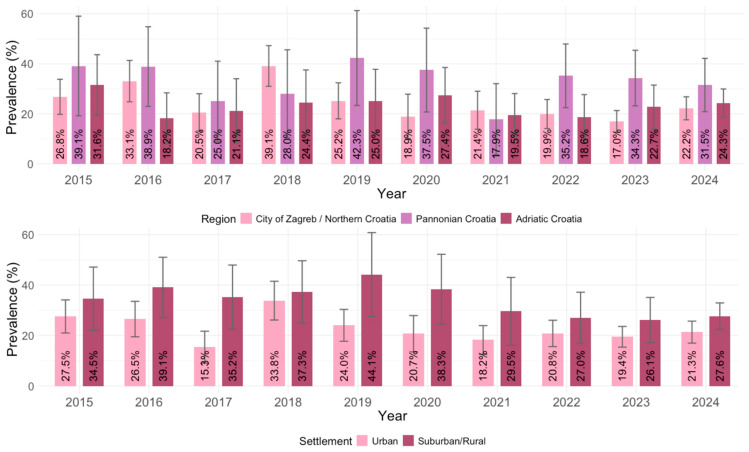
*Toxoplasma gondii* IgG seroprevalence by geographic region and settlement type (% positive with 95% confidence intervals).

Significant regional differences in the IgG seropositivity were observed (*p* = 0.004). The overall IgG seroprevalence rate was higher in Pannonian Croatia (131/394; 33.0%; 95% CI = 28.4–37.6%), than in the City of Zagreb/Northern Croatia (386/1634; 23.7%; 95% CI = 21.6–25.7%) and Adriatic Croatia (178/763; 23.3%; 95% CI = 20.3–26.3%) (Figure 6).

In the seroprevalence analysis according to the settlement, a significantly higher overall IgG seroprevalence was found in residents of suburban/rural regions (250/795; 31.4%; 95% CI = 28.2–34.0%) than in residents of urban regions (445/1994; 22.3%; 95% CI = 20.5–24.1%) (*p* < 0.001). These differences were observed in all tested years (Figure 6).

### 3.4. Risk Analysis for Toxoplasma gondii IgG Seropositivity

**Figure 7:**
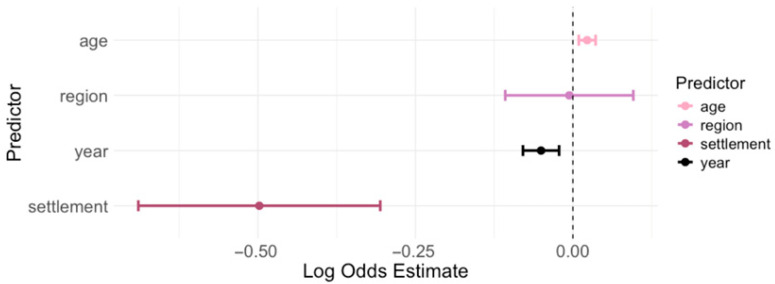
Logistic regression analysis for the risk of *Toxoplasma gondii* IgG seropositivity. A coefficient > 0 means the predictor is associated with increased odds of being IgG-positive, and a coefficient < 0 means decreased odds of being positive. Predictors whose confidence intervals exclude 0 are statistically significant.

The log odds estimates for each predictor in the logistic regression model with the 95% CI are presented in Figure 7 and Table 2.

The results of the logistic regression showed that year of testing, age, and settlement were associated with the risk of IgG seropositivity (Figure 7). For each calendar year, the log odds of being IgG-positive decreased by 0.047 (*p* < 0.001), while for each additional year of age, the log odds increased by 0.021 (*p* < 0.001). Residence in an urban area was associated with lower log odds (*p* < 0.001), whereas the geographic region was not a significant predictor for the log odds of being IgG seropositive (*p* = 0.911). The significant differences in the overall seropositivity among regions (Chi-square test: *p* = 0.004) can be attributed to Pannonian Croatia, where individuals had significantly higher odds of IgG seropositivity compared to the referent region City of Zagreb/Northern Croatia (unadjusted OR = 1.50; 95% CI = 1.15–1.96, *p* = 0.002). Adriatic Croatia did not differ significantly from the City of Zagreb/Northern Croatia in either the unadjusted (*p* = 0.739) or adjusted models (*p* = 0.955). After controlling for age and year, Pannonian Croatia remained a significant predictor (*p* = 0.001), indicating a strong regional effect.

**Figure 8:**
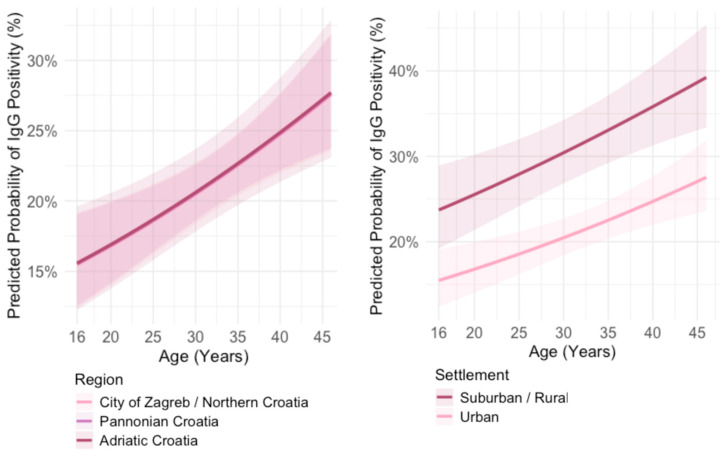
Predicted probability of *Toxoplasma gondii* IgG positivity by age and geographic region (**left**); by age and settlement type (**right**).

The predicted probability for IgG seropositivity in all geographic regions increased with age; however, the difference in probability of being IgG positive did not differ significantly among regions (*p* = 0.911) (Figure 8).

The predicted probability of being IgG positive in all age groups was significantly higher (*p* < 0.001) in participants from suburban/rural settlements. In both urban and suburban/rural settlements, the predicted probability for IgG seropositivity increased with age (Figure 8).

## 4. Discussion

The burden of congenital toxoplasmosis in the EU/EEA is challenging due to several reasons, including variable national surveillance systems and screening practices, underdiagnosis, and lack of standardized data collection [29].

Toxoplasmosis remains a significant concern for childbearing-aged and pregnant women in Europe, with seroprevalence varying notably across countries and regions. While some countries have observed declining trends, others have reported persistently high rates, underscoring the importance of targeted prevention and screening strategies. Determining toxoplasmosis seroprevalence in childbearing-age and pregnant women is critically important for public health measures planning and prevention of congenital toxoplasmosis.

In this study, the overall IgG seropositivity among childbearing-aged and pregnant women was found to be 24.9%, which is lower compared to a Croatian study conducted in 2005–2009 (29.1%) [25]. Many seroepidemiological studies on the prevalence of toxoplasmosis in this risk population group in European countries were conducted in 1990s and 2000s, which showed a wide range of seropositivity rates. Southern and Eastern European countries generally report higher seroprevalence rates than those in Northern and Western Europe. In the 1990s and early 2000s, the highest seropositivity was found in the western countries (31.70–62.79%) [16,30], followed by eastern countries (15.97–55.99%) [18,31], while the lowest seroprevalence was found in northern countries, such as Norway (up to 10%) [32]. Similar regional differences were observed in the late 2000s [13].

More recent studies also showed regional variations in the seroprevalence in Europe: 12.7% in Serbia [33], 17.5% in the Netherlands [34] 24.1% in Kosovo and Metohija [35], 24.2% in France [36], and up to 49.3% in Bulgaria [37].

In addition to variations in seropositivity between countries, regional differences were also observed within the same country. A seroepidemiological study conducted in Central and Southern Italy (2013–2017) included two provinces. The seroprevalence was significantly higher in the province of Bari (Apulia, Southern Italy; 22.4%) than in the province of Siena (Tuscany, Central Italy; 12.4%) [38]. In addition, a study conducted in French pregnant women (2016), the overall seroprevalence was 31.3%. A significant difference was observed by region, with the highest seropositivity in the overseas departments (37.3–76.0%). In mainland France, seroprevalence was highest in the Paris (35.8%) and south-western regions (33.8% and 35.1%, respectively), and lowest in eastern regions (19.1–25.8%) [39].

Regional differences were also observed in Croatia and confirmed to be statistically significant. Residents of Pannonian Croatia showed a significantly higher seroprevalence rate (33.0%) than residents of the City of Zagreb/Northern Croatia (23.7%) and Adriatic Croatia (23.3%). Logistic regression confirmed that these differences in the overall seropositivity among regions can be attributed to Pannonian Croatia, where individuals had significantly higher odds of IgG seropositivity. After controlling for age and year, Pannonian Croatia remained a significant predictor for being IgG seropositive, indicating a strong regional effect. Several factors may contribute to the higher seroprevalence of toxoplasmosis in continental regions, including climate and environment, dietary habits, and agricultural practices. Continental regions have a more humid and temperate climate, which is favorable for the survival of *T. gondii* oocysts in the soil and environment [40]. In contrast, coastal areas with a Mediterranean climate are hotter and drier, which can reduce the survival time of oocysts in the environment, decreasing the probability of transmission. Regarding dietary habits, a higher consumption of undercooked or raw meat, particularly pork, can contribute to the higher seropositivity in continental areas. Coastal regions traditionally consume more fish and seafood, which are not contaminated with toxoplasma, reducing overall risk. Continental counties typically have more extensive livestock farming, including backyard animal husbandry, where sanitation and veterinary control may be limited. Close contact with potentially infected animals and meat processing increases the risk of infection. Contact with infected cats as definite hosts is also a risk factor for the transmission of toxoplasmosis [1]. Outdoor cats in rural continental areas may have more contact with infected prey, increasing environmental contamination with oocysts. Coastal tourist areas may have more controlled animal populations due to public health measures.

A declining trend in the toxoplasma IgG seropositivity over time was observed in Croatia. Seroprevalence rates were higher in 2015–2019 (overall 28.8%, range 21.3–34.8%) than in 2020–2024 (overall 22.6%, range 20.3–25.6%). Analyzing yearly seroprevalence rates, except for 2017, a decreasing trend was observed from 2018 to 2021, with stable rates thereafter. Declining trends in the seroprevalence were also observed in several European countries, including Poland, France, Italy, and Romania. In Poland, a yearly decline in the prevalence of 1.0% was observed. The most important factor contributing to this decline was the significant decrease in seroprevalence among women aged 19 to 29 years [41]. The toxoplasma seroprevalence decreased consistently in France from 26.4% in 2017 to 22.1% in 2023 [36]. A more recent study from Romania showed that *T. gondii* seroprevalence decreased from 43.79% in 2008–2010 to 38.81% in 2015–2018 in both urban and rural areas [42]. Between 2019 and 2023 in Italy, the prevalence of toxoplasmosis remained consistently low at around 11%, with a noticeable decline to 7% in 2023 [43]. In contrast, in Slovakia, the seroprevalence of toxoplasmosis has shown a slightly upward trend (for IgM), remaining stable for IgG seropositivity [44].

There are several possible explanations for the declining trend in toxoplasma seropositivity over the past two decades. Improved food safety and hygiene, including stricter regulations in meat production, may have an impact on reduced contamination [45]. In addition, increased consumption of frozen meat decreases the risk of toxoplasmosis (freezing inactivates tissue cysts). Changes in dietary habits may also influence the transmission of toxoplasmosis. Reduced consumption of raw or undercooked meat, especially traditional dishes such as steak tartare and raw sausages, and increased vegetarianism decreases the risk of *T. gondii* infection [1]. Reduced contact with soil is another possible explanation for a decreasing seroprevalence. Fewer people engage in agriculture or gardening without gloves, which are a common exposure route. Indoor lifestyle reduces the exposure to oocysts from cat feces in soil or sandboxes. Changes in farming practices, such as intensive indoor animal farming, limit exposure of livestock to contaminated soil, feed, or water [46]. In addition, the decreased seroprevalence is at least partly due to the replacement of older, less specific tests with more sensitive and specific serological methods, such as ELISA, immunoblot, and IgG avidity testing.

In Croatia, the overall seroprevalence rates differed between age groups, but without an obvious age-related trend. These differences were significant only in the 2015–2019 period. An age-related increase in toxoplasma seropositivity has been frequently reported. In many countries, seroprevalence increases with age, reflecting cumulative exposure over time [35,36,39,42]. In Bulgaria, the age seroprevalence curve was reverse U-shaped, with the lowest seroprevalence in the 15–19 and >40 year groups (7 and 5%, respectively), while it was highest in the 30–34 year group (37.2%) [37]. A similar age distribution was observed in Serbia, with the highest seropositivity in the 30–34 age group (47.4%) [33].

The results of this study showed a significantly higher overall IgG seroprevalence in residents of suburban/rural regions (31.4%) than in residents of urban regions (22.3%). These differences were observed in all tested years. Rural regions provide more favorable conditions for toxoplasma transmission. Increased environmental exposure due to contact with soil in rural regions may contribute to the seropositivity. People in rural areas are more likely to engage in farming, gardening, or handling soil, increasing the chance of exposure to *T. gondii* oocysts. Cats are more commonly kept outdoors in rural areas, shedding oocysts into the environment. Rural populations consume more home-raised or locally produced meat, which may undergo less rigorous inspection or cooking. Unpasteurized dairy products or untreated water can also be potential sources of infection in some rural settings. Sanitation standards may be less rigorously maintained in rural regions, increasing the risk of environmental contamination. Close contact with livestock (intermediate hosts) increases the risk of infection through occupational exposure or food handling.

This study has some limitations that need to be addressed. The retrospective design of the study based on collected laboratory data limits the availability of detailed information on individual-level risk factors, such as socioeconomic status, occupation or dietary habits, which may affect the variations in seroprevalence. Considering that the study was performed at the national reference institution, those who access private healthcare or even some marginalized groups may have been systematically excluded, potentially introducing selection bias. In addition, the potential for misclassification due to borderline or equivocal serological results exists, especially in the absence of follow-up testing.

## 5. Conclusions

The results of this study indicate that a large proportion of women of childbearing age and pregnant women in Croatia are seronegative for *T. gondii*, making them susceptible to primary infection during pregnancy. Information about the serological status enables the implementation of targeted educational and prevention campaigns. Moreover, early detection of infection allows timely treatment, reduces the risk of transmission, and improves fetal health outcomes. Seroprevalence studies also help monitor shifts in exposure risk over time and the adjustment of strategic priorities.

## Figures and Tables

**Table 1:** Toxoplasma gondii IgG seropositivity by age group and year of testing.

Year	*Toxoplasma gondii* IgG Positive (95%CI)						
	16–20 Yrs	21–25 Yrs	26–30 Yrs	31–35 Yrs	36–40 Yrs	41–45 Yrs	*p* Value
2015 (*n* = 233)	23.5%	32.3%	27.4%	25.8%	32.6%	38.9%	0.863
	(9.6–47.3)	(18.6–49.9)	(17.9–39.6)	(16.6–37.9)	(20.5–47.5)	(20.3–61.4)	
2016 (*n* = 215)	33.3%	15.8%	36.0%	20.9%	34.7%	50.0%	0.098
	(13.8–60.9)	(5.5–37.6)	(24.1–49.9)	(12.9–32.1)	(22.9–48.7)	(29.0–71.0)	
2017 (*n* = 178)	18.8%	6.7%	22.7%	25.6%	18.8%	33.3%	0.591
	(6.6–43.0)	(1.2–29.8)	(12.8–37.0)	(14.9–40.2)	(10.2–31.9)	(13.8–60.9)	
2018 (*n* = 204)	15.4%	47.6%	23.1%	42.3%	21.2%	50.0%	0.026
	(4.3–42.2)	(28.3–67.6)	(12.6–38.3)	(32.0–53.4)	(10.7–37.8)	(29.9–70.1)	
2019 (*n* = 209)	20.0%	28.6%	23.8%	27.5%	25.0%	40.9%	0.741
	(5.7–51.0)	(11.7–54.6)	(13.5–38.5)	(18.4–39.0)	(15.2–38.2)	(23.3–61.3)	
2020 (*n* = 168)	7.1%	23.1%	29.3%	24.2%	33.3%	27.3%	0.584
	(1.3–31.5)	(8.2–50.3)	(17.6–44.5)	(15.2–36.2)	(18.6–52.2)	89.7–56.6)	
2021 (*n* = 220)	9.1%	28.6%	23.8%	18.0%	19.6%	17.6%	0.743
	(1.6–37.7)	(15.3–47.1)	(13.5–38.5)	(10.4–29.5)	(10.7–33.2)	(8.3–33.5)	
2022 (*n* = 305)	24.1%	17.9%	28.1%	18.8%	15.1%	28.6%	0.408
	(12.2–42.1)	(9.0–32.7)	(18.6–40.1)	(11.1–30.0)	(7.9–27.1)	(18.4–41.5)	
2023 (*n* = 447)	12.5%	14.3%	18.7%	19.5%	22.0%	32.3%	0.168
	(4.3–31.0)	(6.7–27.8)	(12.4–27.1)	(13.4–27.6)	(14.7–31.5)	(22.2–44.4)	
2024 (*n* = 610)	11.4%	21.1%	22.4%	25.4%	28.1%	25.7%	0.418
	(4.5–26.0)	(12.5–33.3)	(16.5–29.8)	(19.5–32.5)	(21.1–36.5)	(17.1–36.7)	

**Table 2:** Estimated risk for *Toxoplasma gondii* IgG positivity.

Parameter	Estimated Risk (Log Odds)	Standard Error	*p* Value
Year	−0.050569	0.014658	<0.001
Age	0.022655	0.006810	<0.001
Region	−0.005753	0.051855	0.911
Settlement	−0.497850	0.097975	<0.001

## Data Availability

The original contributions presented in the study are included in the article, further inquiries can be directed to the corresponding author.

## Abbreviations

The following abbreviations are used in this manuscript:

EU/EEA	European Union/European Economic Area
IQR	Interquartile range
NUTS	Nomenclature of Territorial Units for Statistics
CI	Confidence intervals
ELFA	Enzyme-linked fluorescence assay
ELISA	Enzyme-linked immunosorbent assay
